# Understanding pathways to social inequalities in childhood unintentional injuries: findings from the UK millennium cohort study

**DOI:** 10.1186/s12887-019-1514-7

**Published:** 2019-05-15

**Authors:** M. Campbell, E. T. C. Lai, A. Pearce, E. Orton, D. Kendrick, S. Wickham, D. C. Taylor-Robinson

**Affiliations:** 1 Department of Public Health and Policy, Farr Institute, University of Liverpool, Liverpool, L69 3GB UK; 2 University College London Great Ormond Street Institute of Child Health, London, WC1N 1EH UK; 3 Division of Primary Care, School of Medicine, University of Nottingham, Nottingham, NG7 2HA UK

**Keywords:** Unintentional injuries, Inequalities, Socioeconomic, Longitudinal, Cohort, Child health

## Abstract

**Background:**

Childhood unintentional injuries (UI) are common but continue to happen more often to children living in less advantaged socioeconomic circumstances (SEC). Our aim was to explore how early life factors mediate the association between SEC and UIs, using the UK Millennium Cohort Study.

**Methods:**

We calculated risk ratios (RR) and 95% confidence intervals (95%CI) for parental report of UI occurring between age 3 and 5 years, using Poisson regression according to family income as a measure of SEC. We explored potentially mediating pathways by controlling associations between SEC and UI for groups of early life risks in three domains: factors that may influence environmental safety, supervision and the MCS child’s abilities and behaviours.

**Results:**

Twenty eight percent of children had a UI from 3 to 5 years old. Children from the lowest income quintile were more likely to be injured compared to those from the highest (RR 1.20 95%CI 1.05, 1.37). Sequentially controlling for early life factors that may influence environmental safety (RR 1.19 95%CI 1.02, 1.38), then supervision (RR 1.18, 95%CI 1.02, 1.36), and finally adding child’s behaviour and abilities (RR 1.15, 95%CI 1.00, 1.34) into the model reduced the RR by 5, 10 and 25% respectively.

**Conclusions:**

Addressing factors that may influence environmental safety and supervision, and the child’s abilities and behaviours only partly explains the increased UI risk between the highest and lowest income quintiles. Further research is required to explore factors mediating associations between SEC and specific mechanisms and types of injuries.

## Background

Childhood unintentional injuries (UI) are common but their frequency, severity and consequences disproportionately impact on those growing up in more disadvantaged circumstances. [1–4] In England and Wales, death rates of children aged 28 days to 15 years due to UIs were 4.5 times higher from routine or manual worker households compared to those from managerial or professional homes in 2001/03 [5]. Despite a decline in medically attended UI rates over time, the social inequalities gradient has persisted [6].

Underpinning most childhood UI prevention policies, the ‘Haddon Matrix of Injury Occurrence’ [1, 7, 8] categorises most known risk factors into: the host (i.e. in this case the child, including their cognitive and/ or physical characteristics); the physical environment; the social environment at the time of the incident, and the agent of injury defined by the of mechanism of injury [7]. Nearly all injury risk factors are more commonly experienced by children growing up in poverty [9]. Yet, we currently lack the essential understanding of the complex pathways linking adverse social conditions to the heightened risk of UIs in childhood, which are needed in order to develop effective interventions and equitable policies [1].

There are a number of plausible pathways explaining why children growing up in lower income households are more likely to experience UIs [10]. This paper explores three of the most common pathways. First, Children growing up in less advantaged households live in more hazardous environments compared to their more affluent peers (such as less safe housing with a greater likelihood of playing on a street rather than in a garden) [7], potentially explaining their increased risk of UIs during childhood. Second, it is suggested environmental hazards can be mitigated for, if children are supervised adequately and nurtured to develop risk avoidance skills [11, 12]. It is argued that stressors for families living in lower income households may impair supervision and thus, further increase UI risk [11]. Third, some children are at greater risk of a UI, because of their individual abilities and behaviours. Such as ADHD [13], visual impairments [14] and risk-taking behaviours [15] which are more prevalent in lower socioeconomic circumstances.

Using a contemporary, nationally representative sample of children from the UK, we aimed to assess the social patterning of UI in children from 3 to 5 years old. We also examined the extent to which any excess risk in UI for children growing up in disadvantaged circumstances was mediated by potentially modifiable early life factors influencing their environment and supervision, and also measures of the child’s abilities and behaviours.

## Methods

### Design, setting, and data source

We used data from the UK Millennium Cohort Study (MCS), a nationally representative UK birth cohort of 19,250 children born between September 2000 and January 2002, sourced from the U.K. Data Service in 2015. This cohort study used trained interviewers to carry out home-based survey interviews with the main responder, usually the primary carer, about their child and their life. These interviews started when the MCS child was aged nine months, were repeated at 3 years and again at 5 years old. This study uses data on 10,210 children with recorded responses on our primary outcome (UI) and exposure (household income), which are defined further below. The MCS oversampled children living in disadvantaged areas and, in the case of England, areas with high proportions of ethnic minority groups by means of a stratified clustered sampling design [16]. Further information on the cohort and sampling design can be found in the cohort profile [16] or online (www.cls.ioe.ac.uk/mcs). The analysis did not require additional ethical or consent approval [17].

### Primary outcome and exposures

The primary outcome was UIs of any injury type. The main respondent was asked if the child, then aged 5 years old, ‘Ever had an accident and was taken to the doctor, health centre or hospital?’ since the previous survey assessment when the child was aged 3; creating a binary outcome variable (yes, no). Our main exposure of interest and potentially mediating explanatory variables were measured before this at ages 9 months and 3 years, to enable temporal sequencing of the exposure, mediator and outcome measures.

Our primary SEC exposure was equivalised household income (EHI), weighted for the number of adults and dependent children in the household, and divided into quintiles. This was used as a stable measure of early life SEC that preceded the mediator and outcome measures [18].

### Potential confounding factors

We adjusted a number of potential confounders for the exposure and outcome: ethnicity (white, non-white) [19] maternal age at MCS child’s birth (14–19, 20–24, 25–34, 35+ years old) [20], and number of other children in the family at MCS child’s birth (only MCS child, 2–3 children, 4+ children) [21] in our baseline analysis. We also adjusted for the child’s sex, since it is strongly related to our outcome (UIs) [1].

### Early life risk factors (potential mediators)

Based on a literature review we were able to map MCS data to enable us to create categories of early life risk factors (potential mediators) appropriate to three of Haddon’s domains: the child’s environment, supervision and the child’s abilities and behaviours.

#### Factors that may influence environmental safety

Factors captured in the MCS that also provide proxy measures for safety in the child’s physical environments include, a count of responder-reported child safety equipment used from five potential items: car seat, safety gate, fireguard, plug socket covers and smoke alarm(s), measured at MCS child (MCSc) age 9 months (none, one, two, three, four or five items) [22]; responder-reported ‘safe places in your area to play’ measured at MCSc age 9 months (safe, not safe) [22]; interviewer assessed safe in-house environment at MCSc age 3 years, using the short form Caldwell and Bradley’s Home Observation for Measurement of the Environment scale (safe, not safe) [23]; responder-reported not having access to a garden at MCSc age 9 months (yes, no) [24]; interviewer assessed living in an ‘organised house’ or not at MCSc age 3, (very organised, organised, average, disorganised, very disorganised) as levels of household chaos is a recognised risk for childhood UI [25]. We also included a measure related to having household pets at MCSc age 3, for risks related to bites and falls (no pets, pets – including: dogs, cats, other furry animals, birds and other animals such as reptiles) [12, 26]. Household smoking at MCSc age 3, was also considered as it relates to increased risk of burns and household fires (non-smoker, smoker) [27]. Type of childcare was included as a potential measure of care and environmental quality in the case of registered childcare (in parental care, unregistered childcare/other members of the family or friends, registered childcare) [24]. We also included a binary measure of responder-reported feelings of safety in the local area at MCSc age 3 (very safe and fairly safe; neither safe nor unsafe, fairly unsafe and very unsafe) [28].

#### Factors that may influence child supervision

Factors that may influence supervision include supervisor’s mental or physical health, risk taking behaviours, and social support [29, 30]. Relevant factors that were also captured in the MCS include main responder’s level of distress in the last month at MCSc age 3, assessed using the Kessler score for mental distress (normal score range 0 to 14, distressed scores > = 15, 30) main responder’s alcohol unit consumption per week (p/w) at MCSc age 3, (none, 1–5, 6–14, > 14 units p/w) [1]; main responder’s style of parenting relevant to the MCS child, (grouped as either ‘structured parenting style with rules’ combining responses to: firm rules and discipline, firm discipline, plus lots of fun, or ‘unstructured/casual parenting style with rules’ combining responses to: doing my best for the children, lots of fun, have not really thought about it) [31]; social support network measured by family and friends living nearby [32] (neither live nearby, just friends, just family or both) and main responder’s having a limiting long term condition (no, yes) [33].

#### Child’s abilities and behaviours

Factors that provide early life proxy measures for the child’s abilities and behaviours that are also captured in MCS, include: Bracken school readiness (child’s mean school readiness score ≥ 80, not school ready [mean score] from 0 to 79), which measures age-related cognitive ability at MCSc age 3 [9]; socio-emotional development using the total score from the Strength and Difficulties Questionnaire (SDQ) which has been categorised (normal score range 0 to 13, borderline score range 14 to 16, abnormal scores equal to or greater than 17), to assess four domains relating to peer problems, conduct disorders, hyperactivity and emotional problems measured at MCSc age 3, based on activities within the last 6 months [34]; responder-reported concerns about MCSc’s hearing (yes, no) [35] and responder-reported concerns about MCSc’s sight both at age 3 (yes, no) [14].

### Analysis strategy and statistical methods

First, we assessed the prevalence of experiencing one or more UIs according to income. We then undertook a univariable analysis estimating risk ratios (RR) and 95% confidence intervals (95%CI) by Poisson regression for the association between early life risk factors and UI. We then progressed to multivariable analysis, using a complete case sample, whereby variables that were significant at the *P* < 0.1 level in the univariable analysis (likelihood ratio test) [36] were adjusted for in order to assess how this changed the RR for UI comparing lowest to highest income quintile.

Using Haddon’s matrix for injury occurrence to provide our three domain definitions, variables were grouped as blocks of potentially mediating risk factors that may influence (i) environmental safety, (ii) supervision and (iii) factors relating to the child abilities and behaviours. Our approach to assess the impact of these three domains on baseline risk was twofold. First, each domain was added to the baseline model individually to assess their potential isolated impact. Second, each domain was sequentially added to baseline: adjusting for environmental safety first, supervision, and finally child’s abilities and behaviours. The order of adjustment in our sequential model reflected our priori hypotheses about the relationship between these three domains (e.g. environment is potentially driving the association between supervision, child abilities and behaviours, and their UI risk). We also assessed the impact of alternative orders of adjustment. Any observed change in RR was taken to indicate potential mediation [24].

We estimated the change in RRs comparing children in the lowest to the highest income quintiles (the SEC gap) after adjusting for each domain of factors to the model. This was calculated as 100x(adj. Baseline RR - adj. Model RR)/(adj. Baseline RR - 1) [37]. Wald tests were used to assess the significance of individual model parameters. All our analyses were conducted in Stata/SE v.13 (Stata Corporation, College Station, TX, U.S.A.) with survey (svy) commands to account for the sample design and attrition up to age 5.

#### Sensitivity analyses

We repeated the analyses using two alternative measures of childhood SEC, maternal education [38] and also lone parent status [39] both reported at MCSc birth. We repeated the analysis using UIs requiring hospital admission (not admitted, admitted), a more severe outcome for our final model. We undertook multiple imputations by chained equations to explore the impact of missing data in our primary analysis. Missing data ranged from one missing data point (maternal age) to 2719 for the Kessler scale. We imputed missing data for 4152 cases spread across 15 different variables, giving an analytic sample of 14,355. These were created using all variables in the final multivariable regression model including our study outcome (UI reported between 3 and 5 years old), primary exposure (household income quintile) and survey weightings. Twenty imputed datasets were calculated, and estimates were combined using Rubin’s rules [40].

Finally, we also undertook a mediation analysis using counterfactual methods to assess how much of the effect of SEC income on childhood UIs is mediated via the three domains. We estimated the Natural Direct Effect (NDE), Natural Indirect Effect (NIE) and Total Effect (TE), after accounting for potential confounding by known covariates, using the *Medflex* package (2018) in R software. This statistical programme gives us the flexibility to assess the effect of specific causal pathways in order to quantify its contribution to the outcome of interest. Unlike previous mediation methods, this contemporary approach takes into account the possible interactions between multiple mediators when calculating the proportion mediated [41].

## Results

In total, 14,335 singleton children had data on both UI and their household income meeting the inclusion criteria, accounting for 94% of all successful interviews at age 5 years. Of these, 10,210 cases were used in the complete case analysis (Figure 3 in Appendix: sample flow chart). In this total sample, 28% of children (*n* = 4019) experienced at least one UI between the ages of 3 to 5 years old. UIs increase in a dose-response manner as household income decreases. The proportion of children that experienced UIs ranged from 24% in the highest income quintile, to 31% in the lowest income quintile (Table 1).

**Table 1:** Percentage of each variable in household income quintiles using the total cohort sample (*N* = 14,355)

Variables	Income quintile					Total	*P*-value^b^
	First (Highest)	Second	Third	Fourth	Fifth (lowest)	Percent (count)	
*Count of child in each quintile*	*2490*	*2695*	*2771*	*3138*	*3261*	*14,553*	–
Unintentional injuries between 3 and 5 years old	24.0	26.9	28.7	29.4	31.0	28.0 (4019)	< 0.001
Baseline Risks							
MCS child (MCSc) is male, recorded at 9 months old	51.3	50.7	51.7	50.8	50.4	51.0 (7335)	0.09
MCSc from non-white ethnic group recorded at 9 months old	8.0	5.5	8.5	16.6	23.4	12.1 (1919)	< 0.001
Maternal age at birth, recorded at 9 months old							
14–19 years	0.5	1.5	4.2	9.0	23.1	7.7 (1114)	< 0.001
20–24 years	3.0	8.3	16.8	25.8	29.1	16.6 (2551)	
25–34 years	94.6	88.6	78.2	64.0	46.8	74.4 (10494)	
35+ years	2.0	1.6	0.8	1.3	1.0	1.3 (195)	
Number of children in the household at birth (including MCSc) recorded at 9 months old							
MCS lone child	56.8	43.0	40.0	31.6	39.4	42.1 (6008)	< 0.001
2/3 children	41.1	54.3	54.8	56.5	47.9	50.9 (7226)	
4 or more children	2.2	2.7	5.3	11.9	12.7	7.0 (1121)	
Factors That May Influence Environmental Safety							
No safe areas to play, MCSc 9 months old	20.4	25.4	34.1	43.0	51.0	34.8 (5367)	< 0.001
Items of named safety equipment used, MCSc 9 months old (count)^a^							
None	0.4	0.4	0.7	3.5	7.6	2.5 (452)	< 0.001
1 piece	3.5	3.8	5.6	11.4	17.6	8.4 (1280)	
2 pieces	18.8	17.6	19.8	22.7	25.4	20.9 (3081)	
3 pieces	25.9	25.3	26.6	26.1	23.8	25.6 (3739)	
4 pieces	31.0	33.4	31.2	23.8	17.3	27.4 (3724)	
5 pieces	20.3	19.6	16.1	12.4	8.2	15.3 (2075)	
No garden, MCSc age 3	1.5	2.3	4.1	9.8	17.0	6.7 (873)	< 0.001
Type of childcare used, MCSc age 3							
Parent care only	47.8	56.2	67.0	84.6	93.5	69.2 (8858)	< 0.001
Registered childcare (e.g. Ofsted inspected nursery)	19.0	26.2	24.9	12.4	5.1	17.8 (2479)	
Non-registered childcare (e.g. family, friends)	33.2	17.6	8.1	3.1	1.4	13.0 (1575)	
Has a household pets, MCSc age 3	42.5	50.8	54.8	51.8	45.8	49.1 (6029)	< 0.001
Poor home safety (HOME score), MCSc age 3	0.1	1.1	0.9	2.3	3.8	1.6 (191)	< 0.001
Exposed to household smoking, MCSc age 3	5.2	6.9	13.3	24.5	36.8	16.9 (2217)	< 0.001
Responder feels unsafe in area, MCSc age 3	5.4	5.6	10.3	17.0	23.0	12.0 (1656)	< 0.001
Disorganised home environment, MCSc age 3							
Very organised	25.0	22.3	19.3	17.4	15.8	20.1 (2608)	< 0.001
Organised	57.2	56.5	55.6	50.5	48.2	53.7 (6967)	
Average	10.2	11.4	13.5	15.5	16.3	13.3 (1668)	
Disorganised	6.3	8.6	10.0	13.7	16.2	10.8 (1379)	
Very disorganised	1.3	1.2	1.6	2.9	3.5	2.1 (261)	
Factors that may influence supervision							
Primary carer distressed, MCSc age 3	0.7	1.0	2.2	3.4	5.9	2.5 (302)	< 0.001
Primary carer Limiting long term condition, MCSc age 3	15.9	18.5	22.0	26.6	25.4	21.51 (2841)	< 0.001
Primary caregiver alcohol units per week, MCSc age 3							
None	34.5	49.9	62.0	71.5	76.3	58.9 (8947)	< 0.001
1–6 units	43.7	36.2	28.2	19.7	14.5	28.4 (3735)	
7–14 units	18.2	11.4	8.0	6.8	6.3	10.1 (1349)	
15 or more units (exceeding current UK Guidance)	3.6	2.5	1.8	1.9	2.9	2.5 (317)	
Has an unstructured parenting style (e.g. few rules) MCSc age 3	45.3	49.4	57.3	61.9	65.3	55.5 (7068)	< 0.001
Family and friends live nearby MCSc age 3							
Both	40.0	50.8	55.8	52.5	50.7	50.0 (7285)	< 0.001
Family only	5.0	6.8	8.8	9.6	12.4	8.5 (1350)	
Friends only	40.3	30.6	23.8	23.7	21.3	27.9 (3796)	
Neither nearby	14.7	11.8	11.6	14.3	15.6	13.6 (1906)	
Factors relating to the mcs child’s abilities and behaviours							
Score from Strength and Difficulties Questionnaire (SDQ) MCSc age 3, graded as:							
Average	90.0	88.6	81.1	72.8	63.6	79.9 (9607)	< 0.001
Borderline	6.4	7.4	11.0	12.5	14.9	10.2 (1258)	
Below average	3.7	4.1	7.9	14.7	21.6	9.9 (1248)	
Sight concerns, MCSc age 3	5.1	5.8	6.4	6.0	6.1	5.9 (566)	0.19
Hearing concerns, MCSc age 3	5.4	4.0	4.2	4.2	4.0	4.3 (853)	0.52
Child is NOT school ready, MCSc age 3	3.5	5.3	9.4	18.2	26.5	12.1 (1586)	< 0.001

*N* = 14,355 with further missing data for some named variables as: Child: minority/ ethnic *(n* = 1454), Strength and difficulties Questionnaire (SDQ) at age 3 *(n* = 2242), School readiness *(n* = 2712), hearing concerns at 3 yrs old *(n* = 69, sight concern at 3 yrs old *(n* = 59) Maternal age at MCS birth *(n* = 1), Mum has a LLTC at 3 years *(n* = 1471) Levels of distress: Kessler score > 5 *(n* = 2719), Parenting style (*n* = 1936), Alcohol *(n* = 7), Social network*(n* = 18), Number of children in household *(n* = 563), Household pet *(n* = 1392), Household smoking at 3 yrs old *(n* = 1471), No safe places to play at 9 months *(n* = 180), No safety equipment at 9 months *(n* = 4), safe areas to play at 3 yrs *(n* = 2365), Childcare type *(n* = 1443), Responder feels safe *(n* = 1471), disorganised home *(n* = 1472)

^a^ List safety equipment includes [no particular order]: stairgate, fireguard, plug socket covers, car seat, and smoke alarm

^b^ Chi squared test

Table 2 shows the results of the univariable and final multivariable analysis. In our univariable analysis low household income, being male, being white, younger maternal age at MCSc’s birth, more children than the MCSc living in the household, no safe areas to play, not having specified items of safety equipment, using informal childcare, having household pets, exposure to household smoking, responder not feeling safe in their area, living in a disorganised household, main responder higher distress (Kessler scores), main responder having a limiting long term condition, higher SDQ score for socioemotional behavioural difficulties and not being school ready were all associated with an increased RR for UI at *P* < 0.1 (1 d.p.) (Table 2).

**Table 2:** Risk ratios 95% confidence intervals unintentional injuries in highest versus lowest income quintiles analysis

Variables	Total sample (n = 14,355)^a^				FINAL MODEL COMPLETE CASE (*n* = 10,210)^b^		
	Univariable				Multivariable		
	Risk ratio (RR)	95% LCI	95% UCI	P-value	RR	95% LCI	95% UCI
Household Income Quintiles							
First (refer: highest)	Ref	–	–	< 0.001	Ref	–	–
Second	1.12	0.99	1.25		1.08	0.96	1.22
Third	1.19	1.06	1.33		1.11	0.98	1.26
Fourth	1.22	1.07	1.38		1.09	0.95	1.26
Fifth (Lowest)	1.27	1.13	1.43		1.15	0.996	1.34
Baseline Risk Factors							
Child’s sex (Ref: female)	1.22	1.14	1.31	< 0.001	1.22	1.13	1.32
Child ethnic group (Ref: White)	0.74	0.66	0.82	< 0.001	0.79	0.68	0.91
Maternal age at MCS birth							
35+ years	Ref	–	–	< 0.001	Ref	–	–
25–34 years	0.69	0.5	0.93		1.07	0.92	1.25
20–24 years	0.84	0.76	0.93		0.95	0.82	1.11
14–19 years	0.99	0.88	1.11		0.77	0.55	1.09
Number of children in the household at birth (including MCS child)							
MCS lone child	Ref	–	–	0.01	Ref	–	–
2/3 children	1.1	1.03	1.17		1.10	1.01	1.20
4 or more children	1.11	0.99	1.25		1.22	1.04	1.44
Factors That May Influence Environmental Safety							
Safe areas to play at home at 9 months (Ref: No safe areas)	1.06	0.99	1.12	0.08	1.00	0.92	1.08
Items of named safety equipment used at 9 months							
None	Ref	–	–	0.04	Ref	–	–
1 piece	1.13	0.87	1.47		1.15	0.79	1.66
2 pieces	1.28	1.01	1.63		1.25	0.9	1.74
3 pieces	1.32	1.04	1.67		1.36	0.98	1.89
4 pieces	1.34	1.06	1.7		1.36	0.98	1.90
5 pieces	1.33	1.04	1.71		1.36	0.97	1.92
Access to a garden at 9 months (Ref: No access)	1.05	0.91	1.21	0.49	–	–	–
Type of childcare used MCSc age 3							
None (parents look after child)	Ref	–	–	0.09	Ref	–	–
Registered childcare provider	0.91	0.83	1		0.99	0.89	1.10
Non-registered childcare provider (e.g. family, friends)	0.94	0.85	1.04		1.03	0.91	1.17
Household pets MCSc age 3 (ref: No pets)	1.14	1.07	1.21	< 0.001	1.04	0.97	1.12
Household smoking MCSc age 3 (ref: None)	1.15	1.05	1.26	< 0.001	1.01	0.90	1.13
Poor home safety (HOME score) MCSc age 3	1.20	0.93	1.55	0.17	–	–	–
Responder feels unsafe in area MCSc age 3	1.12	1.03	1.22	0.01	1.08	0.98	1.19
Disorganised home environment MCSc age 3							
Very organised	Ref	–	–	< 0.001	Ref	–	–
Organised	1.09	1	1.18		1.05	0.95	1.15
Average	1.15	1.02	1.29		1.11	0.98	1.27
Disorganised	1.16	1.02	1.31		1.06	0.92	1.22
Very disorganised	1.61	1.32	1.95		1.35	1.09	1.67
Factors That May Influence Supervision							
Maternal distress at 3 yrs. (Ref: Kessler < 5)	1.33	1.12	1.59	0.01	1.18	0.94	1.47
Limiting long term condition MCSc age 3 (Ref: none)	1.16	1.07	1.26	< 0.001	1.08	0.98	1.19
Alcohol units per week (MCSc age 3)							
None	Ref	–	–	0.58	–	–	–
1–6 units	0.98	0.92	1.05		–	–	–
7–14 units	1.04	0.93	1.16		–	–	–
15 or more units^+^	1.1	0.92	1.32		–	–	–
Approach to parenting MCSc age 3 (Ref: Unstructured)	1	0.93	1.06	0.71	–	–	–
Family and friends live nearby, MCSc age 3 (Ref: both)							
Both	Ref	–	–	0.19	–	–	–
Family only	1.06	0.95	1.17		–	–	–
Friends only	0.95	0.89	1.02		–	–	–
Neither nearby	1.00	0.91	1.11		–	–	–
Factors Relating To The Mcs Child’s Abilities And Behaviours							
Strength and Difficulties Questionnaire (SDQ), MCSc age 3							
Average	Ref	–	–	< 0.001	Ref	–	–
Borderline	0.06	1.03	1.26		1.05	0.93	1.19
Below average	1.32	1.19	1.46		1.16	1.03	1.32
Sight concerns, MCSc age 3	1.02	0.9	1.15	0.75	–	–	–
Hearing concerns, MCSc age 3	1.08	0.95	1.23	0.24	–	–	–
Child is NOT school ready, MCSc age 3	0.91	0.82	1.00	0.06	1.06	0.96	1.18

^a^when *P* < 0.1 (if rounded 1 d.p.) variables were included in the complete case, multivariable analysis. ^b^ Using sample comprising complete cases for all variables in final multivariable analysis. ^+^ 15 or more units is in excess of UK Guidance for all adult

In the final multivariable model (Table 2) there was no significant association between UI and income. The risk of a UI remained significantly higher for MCSc: with other siblings living at home from birth, living in a very disorganised household, who are male, who are from white ethnic group, and that have below average SDQ scores.

**Fig. 1:**
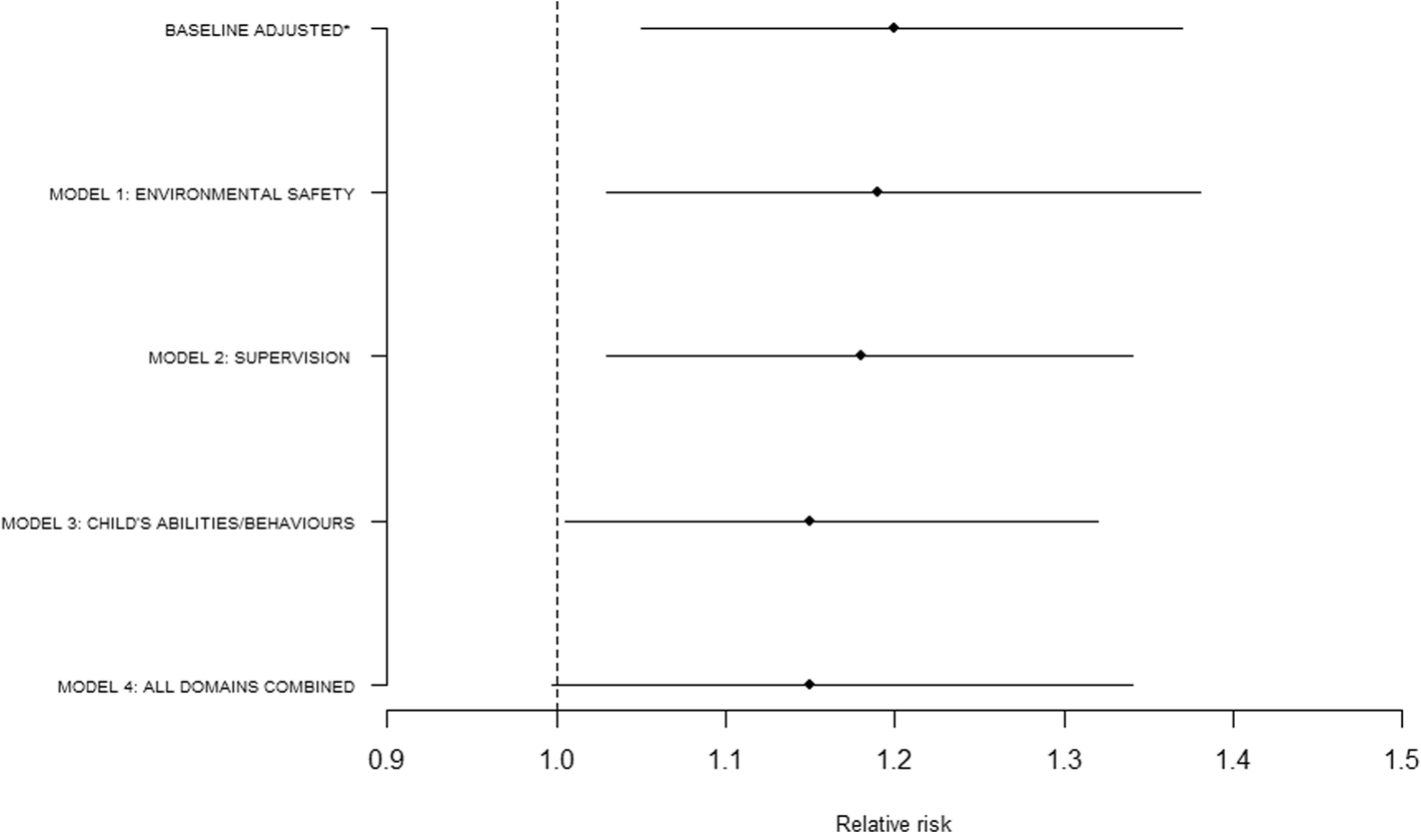
Changes to the injuries Relative Risk by controlling for four separate models of risk factors from the adjusted baseline. *Baseline adjusted for child’s sex and ethnicity, number of children in household and maternal age at birth

We assessed how the baseline RR for UI in the lowest income quintile compared to the highest adjusting for child’s sex and ethnicity, number of children in household and maternal age at birth (aRR 1.20 95%CI 1.05, 1.37) changed after adjusting for each of the UI risk factor domains individually. Adjusting for the environmental safety domain, (e.g. safe areas to play, safety equipment use, childcare type, household pets, in-house smoking, household organization levels and area safety) attenuated the RR by 5% (aRR 1.19 95% CI 1.03 to 1.38). Adjusting for the supervision domain (e.g. primary carer Kessler score and primary carer has a limiting long term condition) attenuated the RR by 10% (aRR 1.18 95%CI 1.03 to 1.34). Adjusting for the child abilities and behaviours domain (e.g. child’s school readiness and SDQ scores) attenuated the RR by 25% (aRR 1.15, 95%CI 1.005, 1.32) (Fig. 1).

**Fig. 2:**
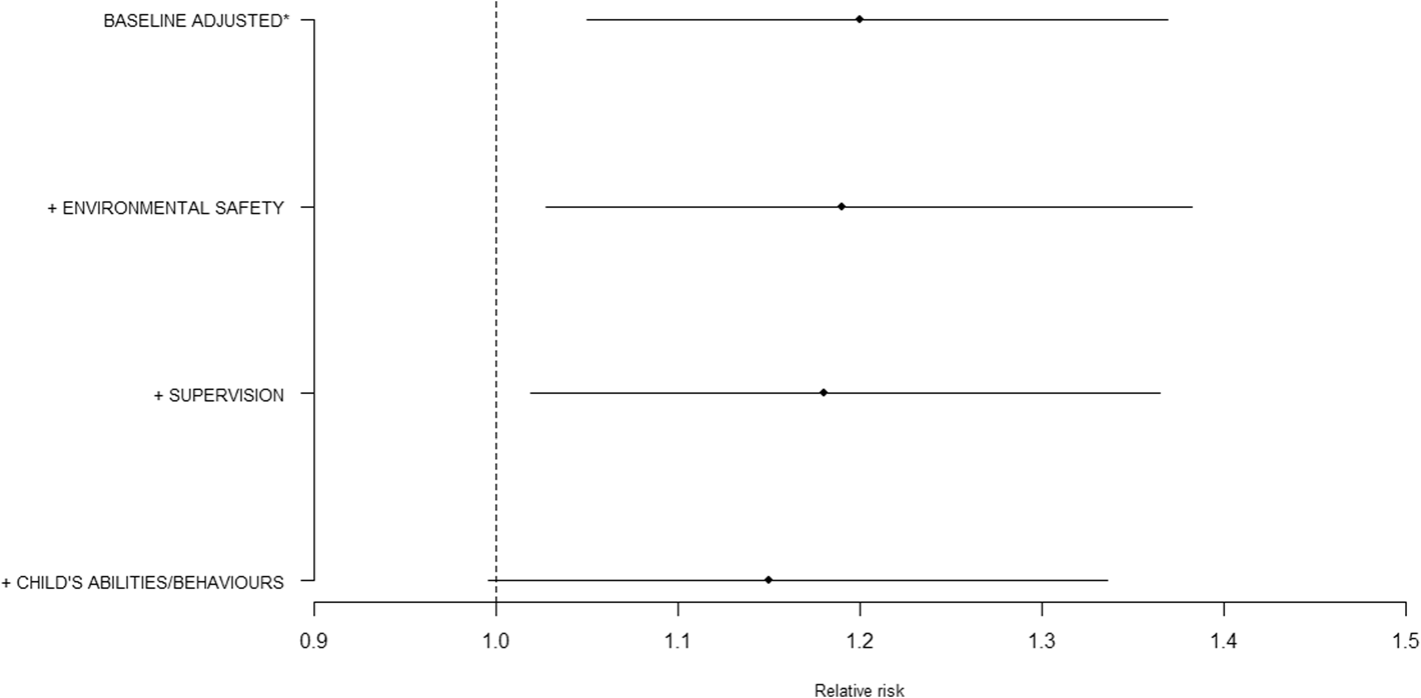
Changes to the injuries Relative Risk by sequentially layering the three domains onto the adjusted baseline. *Baseline adjusted for child’s sex and ethnicity, number of children in household and maternal age at birth

**Fig. 3:**
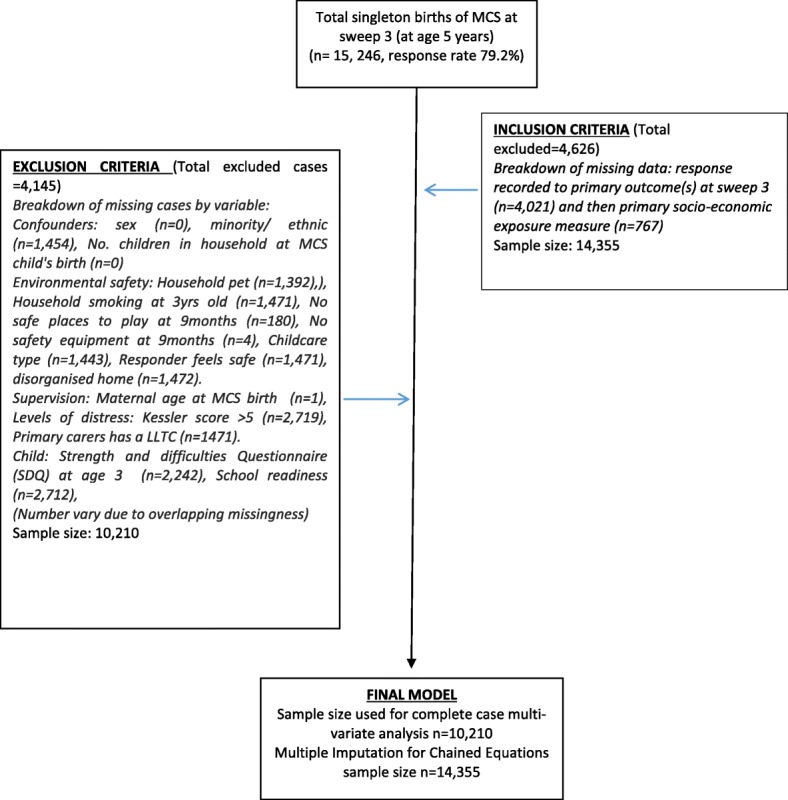
Flow chart of MCS participants with inclusion and exclusion criteria/ numbers from sample

Layering the three domains sequentially, starting with environmental safety, attenuated the baseline risk by 5% (aRR 1.19 95% CI 1.03 to 1.38); then adding the supervision domain attenuated the baseline risk to 10% (aRR 1.18 95%CI 1.02, 1.36), and in our final model (including all three domains) the baseline risk was attenuated to 25% (aRR 1.15 95%CI 0.997 to 1.34), rendering the association between UIs and household income to non-significant (Fig. 2).

### Sensitivity analyses

Our findings were similar when we used lone parent status as an alternative SEC exposure measure and an alternative outcome measure of hospital admissions (Table 3 in Appendix). However, maternal educational, as an alternative measure of SEC did not yield a substantial nor significant reduction in the increase UI risk seen in children with mothers qualified to GCSE D-E/ no qualifications, compared to those with a Degree or higher qualification (Table 3 in Appendix). Repeating the analysis using multiple imputations for missing data showed similar results to our complete case analysis (Table 3 in Appendix).

Our mediation analysis using counterfactual approaches also suggested that the three blocks of mediators (factors that may influence environmental safety, quality of supervision and also the child’s abilities and behaviours), only partially explained income inequalities in UI. Overall 32% of the total effect of income (lowest income quintile versus highest) on childhood UIs is mediated through adjusting for factors that may influence environmental safety, quality of supervision and also the child’s abilities and behaviours, with a total effect of, NDE (1.14 (95% CI 0.98, 1.32)) and NIE (1.06 95% CI 0.98,1.14) (Table 4 in Appendix). Our mediation analysis provides comparable results to our primary analysis and also highlights that a large proportion of the pathways to inequalities are unexplained in this analysis (Table 4 in Appendix).

## Discussion

### Main findings

Using a nationally representative sample of UK children born in 2000–2002, we found more than one in four children (28%) had an unintentional injury (UI) from age 3 to 5 years old. Children from the lowest income households were more likely to have UIs (31%), compared to those living in the highest income homes (24%). We found that the elevated risk in the low income group compared to high was only partially attenuated after adjusting for baseline risks, and potentially mediating factors that may influence environmental safety, supervision, and the child abilities and behaviours.

### Comparison with others findings

The current evidence supports our findings of a social gradient in UIs for preschool children with many of these studies also showing an association with one or more of our exposure domains (e.g. environment, supervision and the child’s abilities and behaviours). [2–4] A systematic review identified 57 empirical studies dated from 1990 to 2009 that explored SEC inequalities for five common UI mechanisms (traffic, drowning, poisoning, burns and falls). [4] The authors concluded that low SEC was associated with increased risk of UI, however the social gradient varied by factors including the environmental settings and the selected measure of SEC. [4] Our study also found the inequalities gap varied by SEC measure used, with lone parent status [versus two parent households] yielding the greatest difference in childhood UI risk, and the least difference seen for maternal educational attainment.

Several studies have sought to better understand the pathways that link SEC to UIs in children. [22, 42, 43] Laflamme and Diderichsen [42] reviewed the literature on traffic injuries in childhood to develop a conceptual framework, based on the Diderichsen model of pathways to social inequalities, [44] that identified potential mechanisms through which social context (e.g. geographical variation in risk), social position (e.g. income, ethnicity and family characteristics), and various exposures (e.g. behaviours) may interact to generate health inequalities. [42] Our analysis shows around 25% of the increased UI risk for children from the lowest income quintile can be explained by early life factors ranging from their social demographics (e.g. family characteristics) to various lifestyle and environmental exposures (e.g. activities and behaviours). This is corroborated by our counterfactual mediation analysis which further suggests that other unexplored pathways to inequalities in UI are likely to exist.

Finally, several factors from across each domain remained significantly associated with increased UI risk, independent of SEC. Similar to other studies, we found an independent increased UI risk in males [15], having below average SDQ scores at 3 years old [45], having a greater number of siblings from birth [21] and living in a very disorganised household at age 3 years [25]. Adding to the on-going debate about ethnicity and UI risk [33], we found that non-white ethnicity was associated with a reduced UI risk. An American based study of preschool-age children also found white children of unemployed mothers living in households needing repair were at higher injury risk than children from other ethnic groups [46].

### Strengths and limitations

We have used a large nationally representative UK cohort that has regularly gathered extensive details on the child, their family and home, and community environments using validated approaches from birth. This enabled us to explore a wide variety of covariates associated with UI risks and broadly reflect the domains of Haddon’s matrix (e.g. the characteristics of the child as the host; in and around the home as the physical environment and factors that may influence supervision reflecting the social environment) for UI from 3 to 5 years old [7].

A limitation of this study is that we did not have sufficiently detailed information about the mechanism and types of the injuries to investigate whether pathways linking adverse social conditions to the heightened risk of UIs vary for specific types of injury. For example, the specific pathways to inequalities in accidental poisoning may differ from those for burns or fractures. Larger studies with more detailed information on injury mechanisms and types are required to examine how potentially mediating pathways might vary by injury characteristics.

The MCS dataset does contain some validated measures of household hazards, quality of supervision and child abilities and behaviours relevant to UI (such as Caldwell and Bradley’s Home Observation for Measurement of the Environment scale) [23] and we have used these in our model. However, we acknowledge these are limited in number. Consequently, we have mainly used indicators that may influence these constructs and that have been used in previous studies [43], but we do not have an assessment of their validity. We judge that our non-validated measures may incompletely capture exposures for UI risk factors, potentially underestimating the proportion mediated by each domain, presenting a non-differential bias [47]. Further studies could build upon our findings using validated measures where these data are available. Equally, we were limited to measures and records at specific time points predetermined by the MCS study. It is essential for our analysis that the mediators occurred before the outcome (UI between 3 and 5 years) event, so our mediator data was collected up to age 3 years.

It is also important to recognise that outcome measures (UI for which medical attention was sought) were reported by the parent, predominantly mothers and may be subject to recall bias [48, 49]. Studies seeking to validate the parent reporting approach have shown it to be more complete at capturing UIs than routine medical notes for more severe injuries, but recall is diminished over time [48]. We accept this study’s two year recall period for childhood UIs, may have led to a conservative prevalence estimate [49], but there is little evidence to suggest that might explain variation in childhood UIs by SEC [48].

An inherent challenge in large cohort studies is missing data. Our main analysis used a complete case sample, whereby individuals with incomplete data on covariates were excluded from the analysis. Sampling and response weights were used to account for the sampling design and attrition. Reassuringly, our sensitivity analysis using multiple imputations produced similar results and conclusions. Finally, our sensitivity analyses using two alternative SEC exposure(s) and a more severe outcome measure (hospital admissions for injury) provide some reassurance about the consistency of the findings from our primary analysis.

## Conclusions

In our analysis, adjusting for a wide range of factors that may influence environment safety, supervision and the MCS child’s abilities and behaviours partially attenuated the excess UI risk experienced by children growing-up in lower income households. Whist this may partially reflect incomplete measurement of potentially mediating pathways, it is likely that there are other explanations for the observed inequality in UI beyond the domains explored in our study. From a health inequalities perspective, the policy and practice implications of our study are that it is unlikely that inequalities in UI for children can be addressed by interventions and policies that only target environmental safety, supervision, or children’s abilities and behaviours. Furthermore, broader policies that aim to improve socio-economic conditions (e.g. increasing household income) are also necessary, and this is particularly important in the context of rising child poverty in the UK, which is likely to increase the risk of a range of adverse outcomes, including UIs.

## Appendix

**Table 3:** Sensitivity analysis for alternative SEC exposures, outcomes and imputed data

Indicators	Adjusted baseline^a^	Model: factors influencing environment	Model: factors influencing environment and supervision	Final model: factors influencing environment and supervision, and child’s abilities and behaviours	Proportion mediated
Alternative measure of SEC exposure					
Maternal education	1.25 (1.10, 1.42)	1.24 (1.09, 1.42)	1.23 (1.09, 1.41)	1.22 (1.07, 1.38)	12%
Lone parent status	1.19 (1.09, 1.30)	1.13 (1.01, 1.26)	1.12 (1.00, 1.25)	1.11 (0.99, 1.24)	42%
Alternative measure of outcome					
Hospital admissions – more severe UIs	2.08 (1.11, 3.89)	1.91 (0 .91, 4.01)	1.93 (0.92, 4.08)	1.79 (0.84, 3.80	27%
Accounting for missing data					
Imputed dataset	1.22 (1.09, 1.36)	1.19 (1.05, 1.35)	1.17 (1.04, 1.33)	1.15 (1.02, 1.30)	32%

^a^ Baseline adjusted for Child’s sex, ethnicity, number of other children at home at birth and mother’s age at MCS child’s birth

**Table 4:** Medflex counter factual mediation analysis for unintentional injuries risk ratios comparing highest versus lowest income quintiles

Models	Effect	RR	95% Lower CI	95% Upper CI	Proportion mediated
MODEL 1: Environmental safety	natural direct effect	1.17	1.01	1.37	9.80%
	natural indirect effect	1.02	0.94	1.09	
	total effect	1.19	1.05	1.36	
MODEL 2: Child supervision	natural direct effect	1.17	1.04	1.33	12.7%
	natural indirect effect	1.02	1.00	1.04	
	total effect	1.20	1.05	1.36	
MODEL 3: Child’s ability and behaviours	natural direct effect	1.15	1.01	1.30	26.3%
	natural indirect effect	1.05	1.02	1.07	
	total effect	1.20	1.05	1.36	
MODEL 4: All three above domains	natural direct effect	1.14	0.98	1.32	31.6%
	natural indirect effect	1.06	0.98	1.14	
	total effect	1.20	1.05	1.37	

**Table 5:** Complete case analysis for variables used in the final model^**b**^

Variables	Complete Case (*n* = 10,210)^b^					
	Univariable^a^			Multivariable		
	RR	95% LCI	95% UCI	RR	95% LCI	95% UCI
Household Income Quintiles						
First (refer: highest)	Ref	–	–	Ref	–	–
Second	1.12	0.99	1.25	1.08	0.96	1.22
Third	1.19	1.7	1.33	1.11	0.98	1.26
Fourth	1.22	1.08	1.38	1.09	0.95	1.26
Fifth (Lowest)	1.28	1.13	1.44	1.15	0.996	1.34
Baseline Risk Factors						
Child’s sex (Ref: female)	1.23	1.14	1.33	1.22	1.13	1.32
Child ethnic group (Ref: White)	0.78	0.68	0.9	0.79	0.68	0.91
Maternal age at MCS birth						
35+ years	Ref	–	–	Ref	–	–
25–34 years	1.07	0.92	1.24	1.07	0.92	1.25
20–24 years	0.92	0.82	1.05	0.95	0.82	1.11
14–19 years	0.76	0.54	1.06	0.77	0.55	1.09
Number of children in the household at birth (including MCS child)						
MCS lone child	Ref	–	–	Ref	–	–
2/3 children	1.11	1.02	1.2	1.1	1.01	1.2
4 or more children	1.25	1.08	1.43	1.22	1.04	1.44
Factors That May Influence Environmental Safety						
Safe areas to play at home at 9 months (Ref: No safe areas)	1.06	0.98	1.14	1	0.92	1.08
Items of named safety equipment used at 9 months						
None	Ref	–	–	Ref	–	–
1 piece	1.11	0.76	1.61	1.15	0.79	1.66
2 pieces	1.18	0.84	1.66	1.25	0.9	1.74
3 pieces	1.28	0.92	1.79	1.36	0.98	1.89
4 pieces	1.28	0.91	1.79	1.36	0.98	1.9
5 pieces	1.27	0.90	1.8	1.36	0.97	1.92
Access to a garden at 9 months (Ref: No access)	–	–	–	–	–	–
Type of childcare used at 3 yrs. old						
None (parents look after child)	Ref	–	–	Ref	–	–
Registered childcare provider	0.94	0.85	1.04	0.99	0.89	1.1
Non-registered childcare provider (e.g. family, friends)	0.91	0.81	1.02	1.03	0.91	1.17
Household pets at 3 yrs. old (ref: No pets)	1.09	1.02	1.17	1.04	0.97	1.12
Household smoking at 3 yrs. old (ref: None)	1.14	1.03	1.26	1.01	0.9	1.13
Poor home safety (HOME score) at 3 yrs. old	–	–	–	–	–	–
Responder feels unsafe in area at 3 yrs. old	1.17	1.06	1.29	1.08	0.98	1.19
Disorganised home environment at age 3 years						
Very organised	Ref	–	–	Ref	–	–
Organised	1.08	0.98	1.19	1.05	0.95	1.15
Average	1.19	1.05	1.36	1.11	0.98	1.27
Disorganised	1.18	1.03	1.35	1.06	0.92	1.22
Very disorganised	1.55	1.25	1.93	1.35	1.09	1.67
Factors That May Influence Supervision						
Maternal distress measured MCSc age 3 (Ref: Kessler < 5)	1.36	1.09	1.68	1.18	0.94	1.47
Limiting long term condition MCSc age 3 (Ref: none)	1.12	1.02	1.24	1.08	0.98	1.19
Alcohol units per week MCSc age 3						
None	–	–	–	–	–	–
1–6 units	–	–	–	–	–	–
7–14 units	–	–	–	–	–	–
15 or more units^c^	–	–	–	–	–	–
Approach to parenting MCSc age 3 (Ref: Unstructured)	–	–	–	–	–	–
Family and friends live nearby, MCSc age 3 (Ref: both)						
Both	–	–	–	–	–	–
Family only	–	–	–	–	–	–
Friends only	–	–	–	–	–	–
Neither nearby	–	–	–	–	–	–
Factors Relating To The Mcs Child’s Abilities And Behaviours						
Strength and Difficulties Questionnaire (SDQ), MCSc age 3						
Average	Ref	–	–	Ref	–	–
Borderline	1.12	0.99	1.26	1.05	0.93	1.19
Below average	1.3	1.16	1.46	1.16	1.03	1.32
Sight concerns, MCSc age 3	–	–	–	–	–	–
Hearing concerns, MCSc age 3	–	–	–	–	–	–
Child is NOT school ready, MCSc age 3	0.85	0.76	0.94	1.06	0.96	1.18

^*a*^*when P* < 0.1 (if rounded 1 d.p.) *variables were included in the complete case, multivariable analysis*

^*b*^ *Using sample comprising complete cases for all variables in final multivariable analysis*

^*c*^ *15 or more units is in excess of UK Guidance for all adults*

## Abbreviations

(N=) or (n=): (N = denominator number/ count) (n = numerator number or count)
aRR: adjusted Risk Ratio
MCS: Millennium Cohort Study
MCSc: Millennium Cohort Study Child
MI: Multiple Imputations
NDE: Natural Direct Effect shown from the Counter Factual Model
NIE: Natural Indirect Effect shown from the Counter Factual Model
RR: Risk Ratio
SDQ: Strength and Difficulties Questionnaire
SEC: Socio-Economic Circumstances
TE: Total Effect shown from the Counter Factual Model
U.S.: United States
UI: Unintentional Injuries
U.K.: United Kingdom
